# Validation of the new pathology staging system for progressive supranuclear palsy

**DOI:** 10.1007/s00401-021-02298-z

**Published:** 2021-03-28

**Authors:** Mayen Briggs, Kieren Simon James Allinson, Maura Malpetti, Maria Grazia Spillantini, James Benedict Rowe, Sanne Simone Kaalund

**Affiliations:** 1 Cambridge University Hospitals NHS Foundation Trust and the Cambridge Brain Bank, Cambridge, CB2 2QQ UK; 2 Department of Clinical Neurosciences, University of Cambridge, Cambridge Biomedical Campus, Cambridge, CB2 0SZ UK; 3 Cambridge Centre for Parkinson-Plus, University of Cambridge, Cambridge, UK; 4 Department of Clinical Neurosciences, University of Cambridge, Cambridge Biomedical Campus, Clifford Allbutt Building, Hills Road, Cambridge, CB2 0AH UK; 5 Medical Research Council Cognition and Brain Sciences Unit, University of Cambridge, Cambridge, CB2 7EF UK

Progressive supranuclear palsy (PSP) is a neurodegenerative disorder associated with neuroglial accumulation of 4-repeat tau protein [2]. Kovacs et al. [1] have recently proposed a new semi-quantitative six-stage system to categorise the severity of PSP pathology. Importantly, the system reduces reliance on regions with high risk of concomitant pathology and focusses on cell type-specific tau-pathology.

Here, we test the new PSP pathology staging system in an independent series of 35 PSP cases and test the potential association between pathology stage and clinical severity at death. We include tissue from 35 people with a clinical diagnosis of PSP (including *N* = 25 with Richardson’s syndrome and *N* = 10 with other phenotypes; Movement Disorder Society 2017 criteria; Supplementary table 1, online resource). Donors had attended longitudinal clinical studies at the Cambridge Centre Parkinson-plus including assessment of clinical severity by the PSP rating scale (PSPRS) and cognitive performance by the revised Addenbrooke’s Cognitive Examination (ACE-R). The left brain hemisphere was available for pathological evaluation (Supplementary methods, online resource) and following the guidelines from Kovacs et al. we rated regional tau cytopathology focussing on astrocytic tau inclusions in striatum (STR), frontal and occipital cortices, and neuronal and oligodendroglia tau inclusions in globus pallidus (GP), subthalamic nucleus (STN), and cerebellum.

First, we selected ten random cases and, in each area, two authors independently rated tau pathology following the new staging system as described by Kovacs et al. [1]. The raters were in agreement in just 45/60 regions (75%). Pallidum, cerebellum and occipital lobe had high agreement (≥ 8/10), STN and frontal cortex intermediate (7/10), while STR had low agreement (4/10). This discrepancy was attributed to the lack of operational criteria and individual interpretations of the staging system ratings for each area, confounded by marked differences between regions in the absolute numbers of immunoreactive cells per field. We therefore formulated operational criteria with region-specific thresholds see Fig. 1c and Supplementary Fig. 1 (online resource) for a visual guide. With the new operational criteria the inter-rater agreement increased to 88% (52/59 regions), with high agreement for GP, STR, frontal cortex, occipital and cerebellum (9/10) and intermediate for STN (7/9). Integrating these operational criteria to the new staging system, 91% (32/35) of cases fitted readily into one of the six stages (Fig. 1a), including 9/10 of the cases with non-Richardson’s phenotype (Fig. 1d and e).

**Fig. 1:**
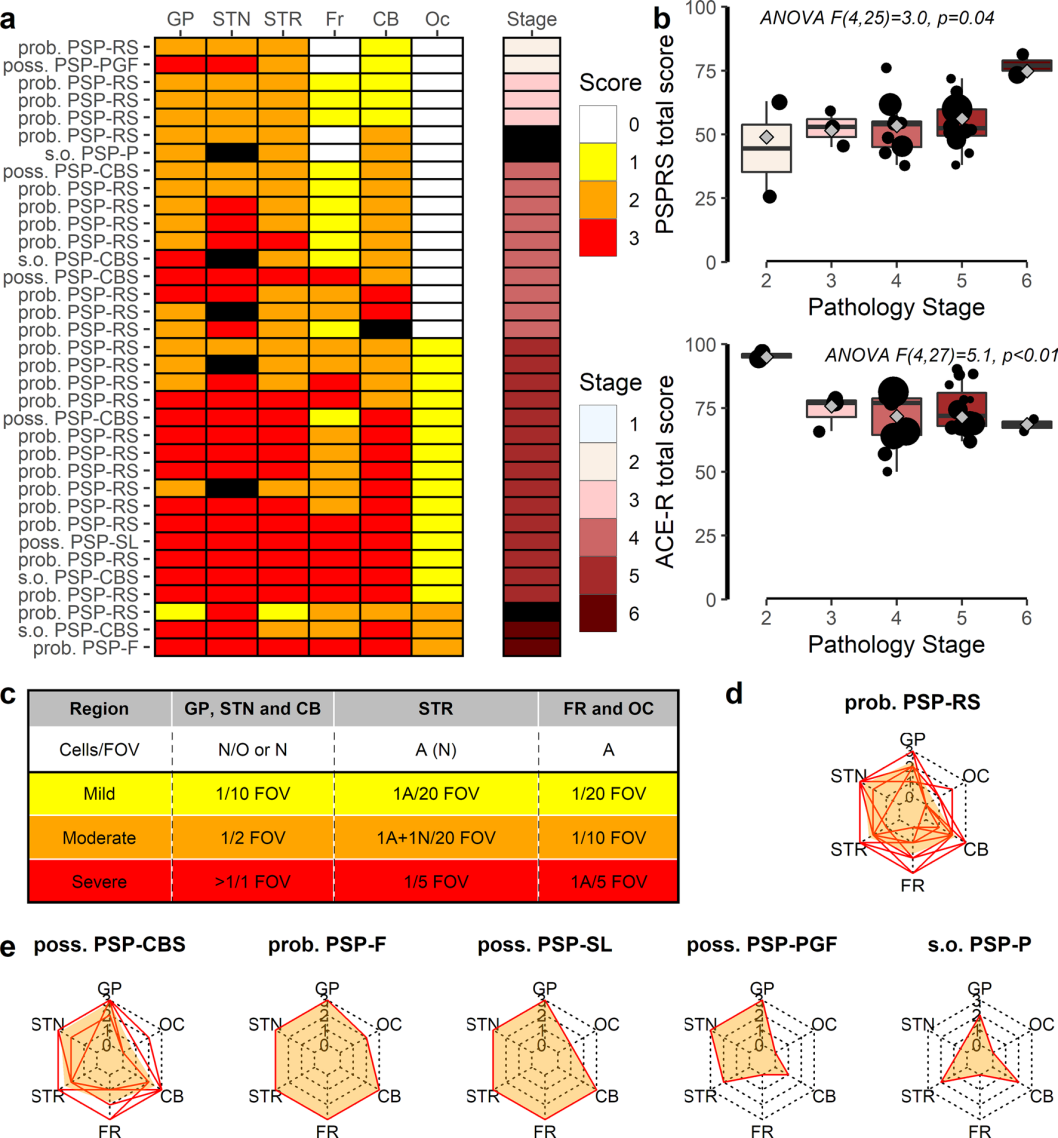
Validation of pathology staging system for PSP. **a** Regional pathology scores (white to red) and pathology staging (light blue to brown). Black tiles indicate a missing region/no stage. Last diagnosis based on the movement disorder criteria is shown to the left of the tiles. **b** Pathology stage was correlated with clinical severity PSPRS and ACE-R. Individual data points are shown with black dots, boxplots show median, first and third quartiles, extending whiskers to 1.5*interquartile range. Grey diamonds show the weighted group means, and the size of the points are proportional to the weight in the analysis. **c** Guide for tau pathology rating. **d** and **e** Cases with subcortical phenotypes, PSP-P and PSP-PGF, showed minimal pathology. Red lines show individual profiles of regional pathology and orange fill the average group profiles. Striped lines show concentric hexagons representing no to severe (0–3) pathology. *PSP* progressive supranuclear palsy, *prob.* probable, *poss.* possible, *s.o* suggestive of, *RS* Richardson’s syndrome, *CBS* corticobasal syndrome, *SL* speech/language variant, *F* frontal, *PGF* progressive gait freezing, *P* parkinsonism, *GP* globus pallidus, *OC* occipital cortex, *FR* frontal cortex, *CB* cerebellum, *STR* striatum, *STN* subthalamic nucleus, *PSPRS PSP* Rating Scale, *ACE-R* revised Addenbrooke’s Cognitive Examination, *FOV* field of view under 40 × objective, *A* tufted astrocytes, *N* neuronal tangles and tau positive threads, *O* coiled bodies, *n.s.* not significant, *p* > 0.05

We then tested whether the pathology staging was associated with demographic, age and clinical severity. There was no significant association between the pathological stages and age or symptom duration (Kruskal Wallis, *p* > 0.05). The interval between death and last assessment of disease severity (PSPRS and ACE-R) varied from 24 days to 35 months. To account for the differences in interval between testing and death we took two approaches (1) weighting the analysis for this time interval (Clinical score ~ PSP pathology stage|weight = 1/interval between assessment and death; Fig. 1b) and, (2) imputing the PSPRS and ACE-R scores at death from longitudinal assessments (imputed score ~ PSP pathology stage; Supplementary Fig. 2a, b and 3, 4, online resource). There was a significant association between pathology stage and PSPRS, both when weighting for time between last testing and death (*F*(4,25) = 30.0, *p* = 0.036) and using imputed PSPRS scores (*F*(4,27) = 2.8, *p* = 0.045). There was a significant association between pathology stage and cognitive deficit, when weighting the analysis for time between testing and death (*F*(4,27) = 5.09, *p* = 0.0035), but not using imputed ACE-R scores (*F*(4, 27) = 2.43, *p* = 0.07). Given the small group sizes for stages 2, 3 and 6, post hoc analysis were not performed.

Overall, our study supports the validity of the proposed PSP pathology staging system proposed by Kovacs et al. [1], being easy to implement in the day-to-day neuropathological evaluation (and retrospectively) as the regions required are routinely sampled for the pathological diagnosis of neurodegenerative disease. The proposed PSP staging schema is applicable across the spectrum of clinical PSP subtypes with > 90% of cases fulfilling staging criteria. In addition to the written description provided by Kovacs et al. we provide region-specific quantitative criteria along with a visual guide for the rating of tau pathology. Together with high compliance with the staging scheme, our findings suggest that the sequential distribution of tau pathology is associated with progressive clinical severity in PSP.

## Supplementary Information

Below is the link to the electronic supplementary material.

Supplementary file1 (PDF 99 KB)

Supplementary file2 (PDF 1261 KB)

Supplementary file3 (PDF 109 KB)

## Data Availability

The datasets used are available from the corresponding author on reasonable request.
