# Supplementary material for: Validation of the new pathology staging system for progressive supranuclear palsy
Source: Acta Neuropathol. 2021 Mar 28;141(5):787–9. doi: 10.1007/s00401-021-02298-z (PMC8043892; doi:10.1007/s00401-021-02298-z)
Supplement: Supplementary file 1 — Supplementary file1 (PDF 99 KB) [file 401_2021_2298_MOESM1_ESM.pdf]

Supplementary Table

Title

Validation of the new pathology staging system for progressive supranuclear palsy

Authors

Mayen Briggs[1], Kieren SJ Allinson[1], Maura Malpetti[2,3], Maria Grazia Spillantini[2], James Benedict Rowe[2,3,4], Sanne Simone Kaalund[2,3]

[1] Cambridge University Hospitals NHS Foundation Trust and the Cambridge Brain Bank, CB2 2QQ

[2] Department of Clinical Neurosciences, University of Cambridge, Cambridge Biomedical Campus, Cambridge, CB2 0SZ

[3] Cambridge Centre for Parkinson-plus, University of Cambridge

[4] Medical Research Council Cognition and Brain Sciences Unit, University of Cambridge, UK, CB2 7EF

Corresponding author

Address: Department of Clinical Neurosciences, Clifford Allbutt Building, Hills Road, CB2 0AH, Cambridge, United Kingdom

Telephone: +44 1223762076

Email: ssk42@medschl.cam.ac.uk

Supplementary Table 1 Demographic and clinical summary statistics of cases

Clinical Diagnosis		Sex	Age at death (years)		Symptom Duration (years)		Interval PSPRS - death (months)		Last PSPRS total		Imputed PSPRS at death		Interval ACE-R - death (months)		Last ACE-R total		Imputed ACER at death	
	n	M/F	Mean	SD	Mean	SD	Mean	SD	Mean	SD	Mean	SD	Mean	SD	Mean	SD	Mean	SD
**prob. PSP-RS**	25	13/12	74.5	7.6	7.6	3.8	12.2	8.9	52.2	10.0	58.2	8.2	10.3	7.7	70.3	16.6	76.4	8.3
**poss. PSP-CBS**	3	1/2	79.2	0.6	4.7	1.2	8.0	2.0	47.7	4.2	55.7	1.1	3.7	3.8	70.7	7.6	82.0	2.2
**s.o. PSP-CBS**	3	2/1	76.2	3.7	6.8	2.3	26.0 *	-	64.0 *	-	73.0	-	37.0	26.3	82.3	9.8	71.1	-
**poss. PSP-SL**	1	-/1	78.4	-	8.8	-	35.0	-	57.0	-	69.2	-	23.0	-	72.0	-	81.5	-
**prob. PSP-F**	1	1/-	75.4	-	8.4	-	9.0	-	81.0	-	71.8	-	27.0	-	66.0	-	69.2	-
**poss. PSP-PGF**	1	1/ -	75.2	-	4.6	-	6.0	-	26.0	-	27.8	-	6.0	-	97.0	-	93.9	-
**s.o. PSP-P**	1	- /1	75.2	-	2.8	-	*	-	*	-	-	-	28.0	-	50.0	-	53.1	-

*Btw. – between, PSPRS – progressive supranuclear palsy rating scale, ACE-R – revised Addenbrooke's Cognitive Examination, M – male, F- female, PSP – progressive supranuclear palsy, Prob.- probable, poss. – possible, s.o. –suggestive of, RS- Richardson's syndrome, CBS – corticobasal syndrome, SL – speech/language variant, F – frontal, PGF – progressive gait freezing, P - parkinsonism. * missing PSPRS assessment*
