# Supplementary material for: Validation of the new pathology staging system for progressive supranuclear palsy
Source: Acta Neuropathol. 2021 Mar 28;141(5):787–9. doi: 10.1007/s00401-021-02298-z (PMC8043892; doi:10.1007/s00401-021-02298-z)
Supplement: Supplementary file 2 — Supplementary file2 (PDF 1261 KB) [file 401_2021_2298_MOESM2_ESM.pdf]

Supplementary Figures

Title

Validation of the new pathology staging system for progressive supranuclear palsy

Authors

Mayen Briggs[1], Kieren SJ Allinson[1], Maura Malpetti[2,3], Maria Grazia Spillantini[2], James Benedict Rowe[2,3,4], Sanne Simone Kaalund[2,3]

[1] Cambridge University Hospitals NHS Foundation Trust and the Cambridge Brain Bank, CB2 2QQ

[2] Department of Clinical Neurosciences, University of Cambridge, Cambridge Biomedical Campus, Cambridge, CB2 0SZ

[3] Cambridge Centre for Parkinson-plus, University of Cambridge

[4] Medical Research Council Cognition and Brain Sciences Unit, University of Cambridge, UK, CB2 7EF

Corresponding author

Address: Department of Clinical Neurosciences, Clifford Allbutt Building, Hills Road, CB2 0AH, Cambridge, United Kingdom

Telephone: +44 1223762076

Email: ssk42@medschl.cam.ac.uk

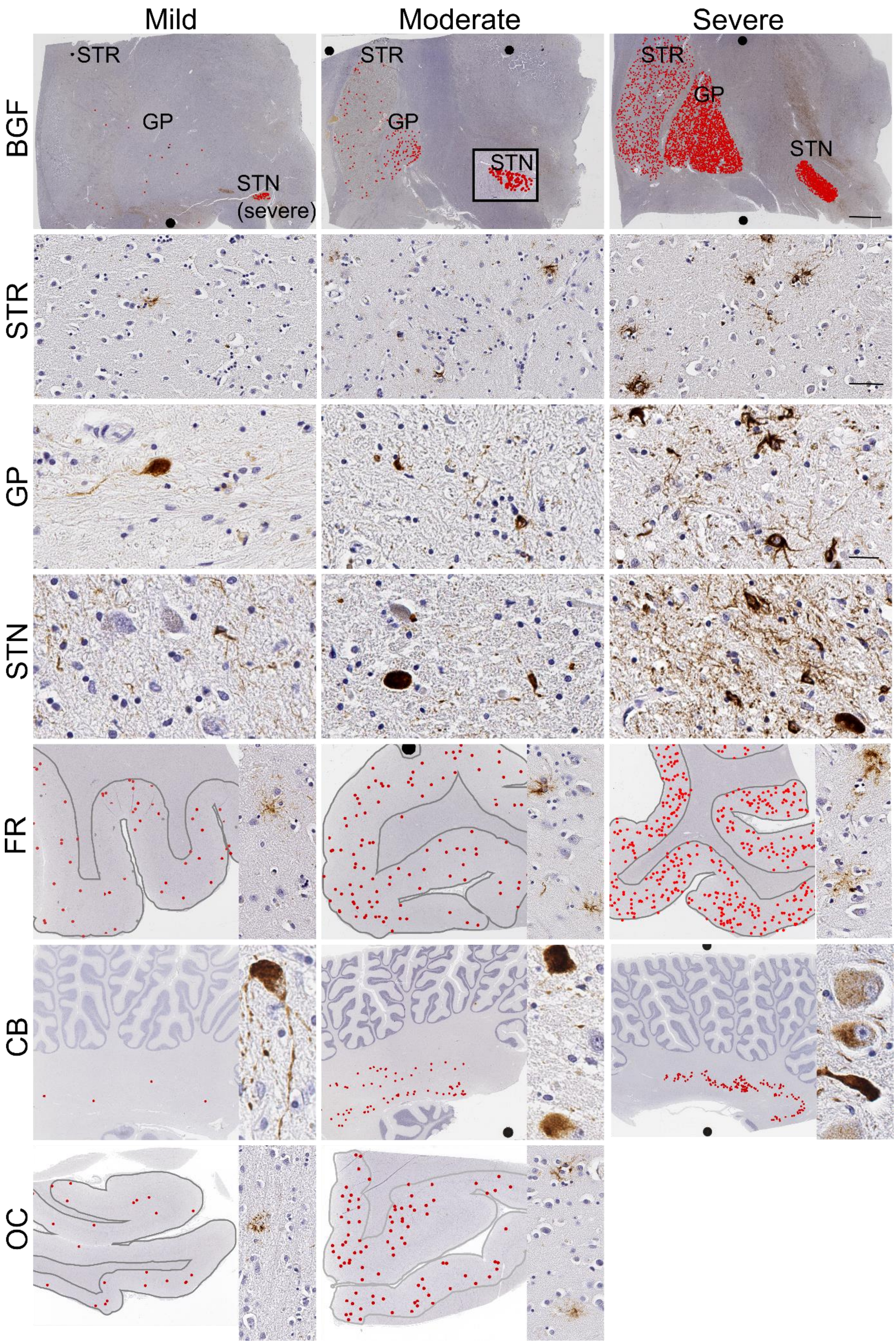

Mild
Moderate
Severe
BGF
STR
GP
STN
FR
CB
OC
STR
GP
STN
(severe)
STR
GP
STN
STR
GP
STN

Supplementary fig. 1 *Visual guide to rating of PSP tau pathology. Micrographs of sections stained for hyperphosphorylated tau (AT8) showing the regional density of tau-aggregates (red points) at low magnification (0.3X, scale bar 2mm), and at higher magnifications; STR, FR and OC (20x, scale bar 50 µm), GP, STN and CB (40x, scale bar 20 µm). Left column shows regions rated "mild" – with the exception of STN where we did not have an example of "mild" pathology, thus the STN on the low magnification has "severe" pathology, and at high magnification we chose an area of STN with close to mild pathology from a STN rated "moderate". The middle panel shows regions rated "medium" – on the low magnification, an example of "moderate" STN is superimposed on the STN for the given section which had a rating of "severe". Right column shows regions rated "severe" – with the exception of occipital lobe as we did not have an example of "severe" occipital pathology. For cerebellum only pathology in the dentate nucleus, not white matter, is represented. Large black dots on the low magnification micrographs are image artefacts from the digital slide scanning. BGF – basal ganglia, STR – striatum, GP – globus pallidus, STN – subthalamic nucleus, FR – middle frontal gyrus, OCl – primary visual cortex*

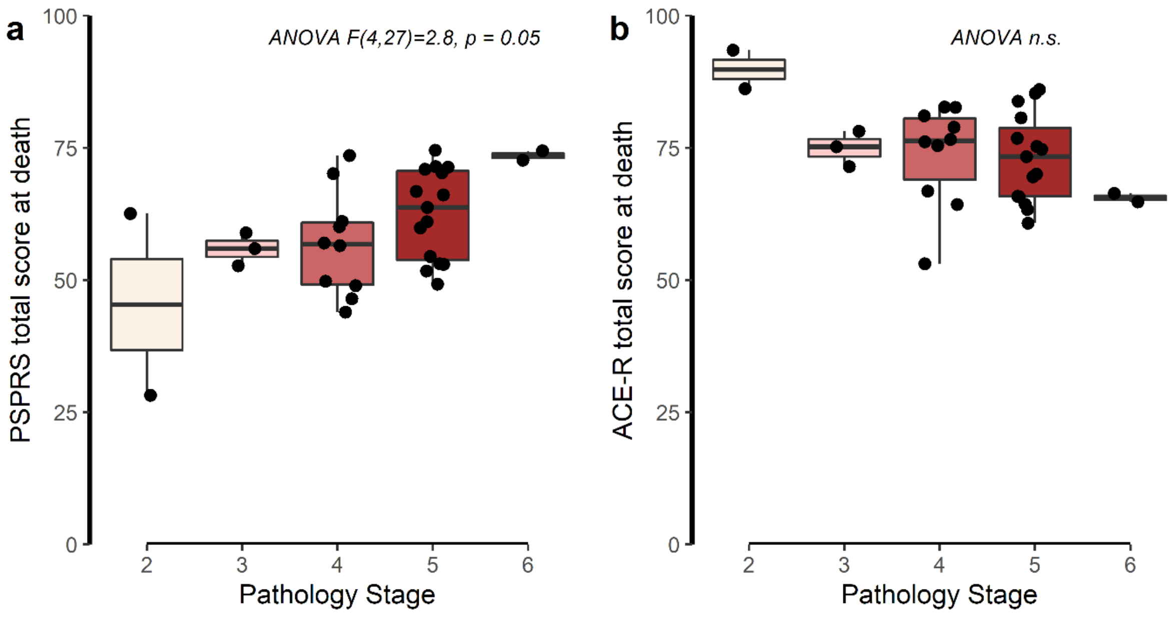

Supplementary fig. 2 *PSPRS and ACE-R according to pathology stage. a and b, we imputed the values for PSPRS and ACE-R at death for each individual, black points. There was a significant effect of pathology stage and the imputed values for PSPRS, but not for ACE-R. PSPRS – Progressive Supranuclear Palsy Rating Scale, ACE-R – revised Addenbrooke's Cognitive Examination, n.s. – not significant, p > 0.05*

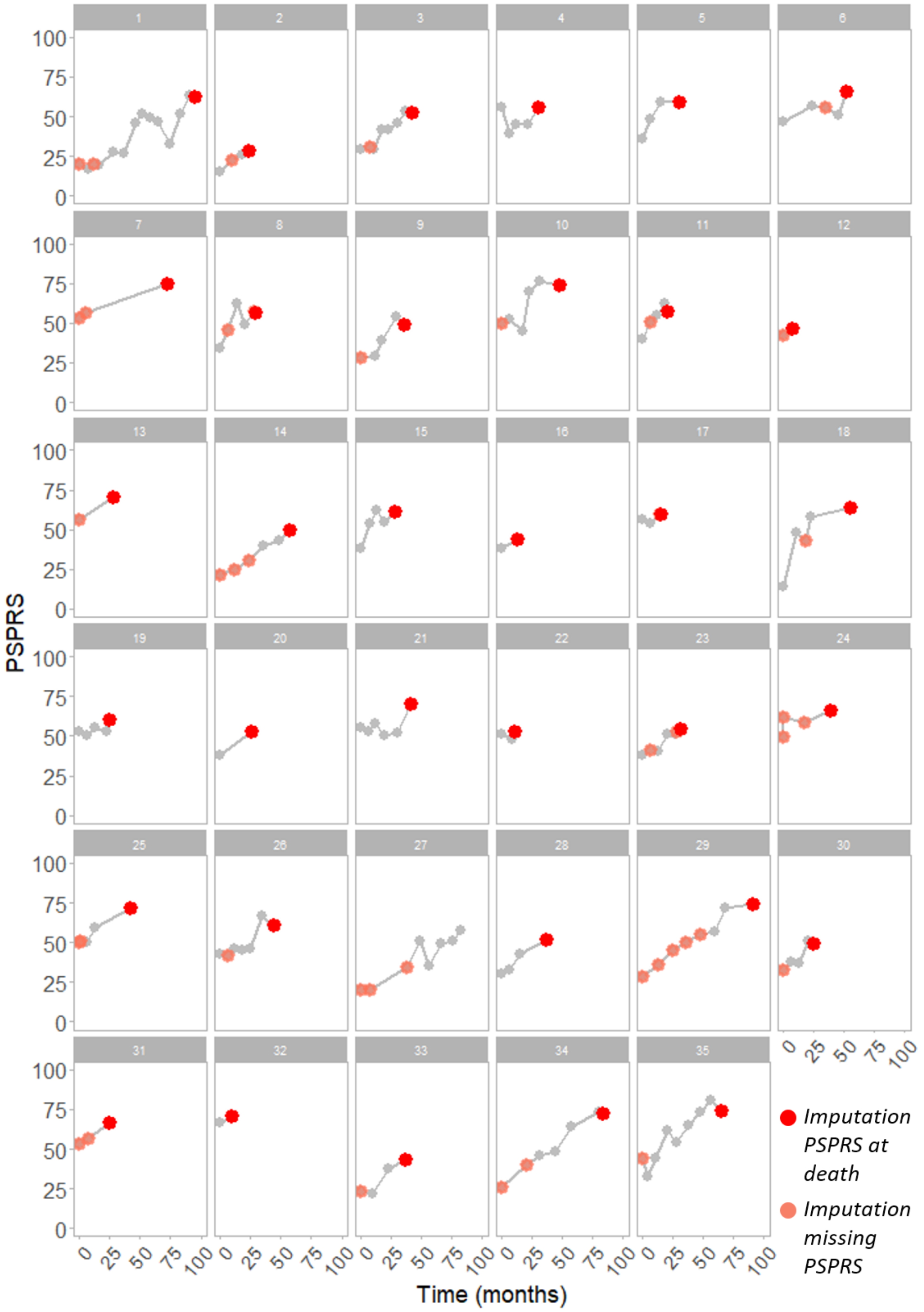

Supplementary fig. 3 *Longitudinal and imputed PSPRS for individual PSP patients. PSPRS and ACE-R accessed at visits to the clinic (grey points) were used to impute PSPRS values at death (red points) and at visits where only ACE-R were measured (pink points) for each of the 35 PSP patients. The x-axis show time from the first visit (0) to death in months. The order of patients (1-35) is commensurate with that of Figure 2. ACE-R – revised Addenbrooke's Cognitive Examination revised, PSPRS – Progressive Supranuclear Palsy Rating Scale*

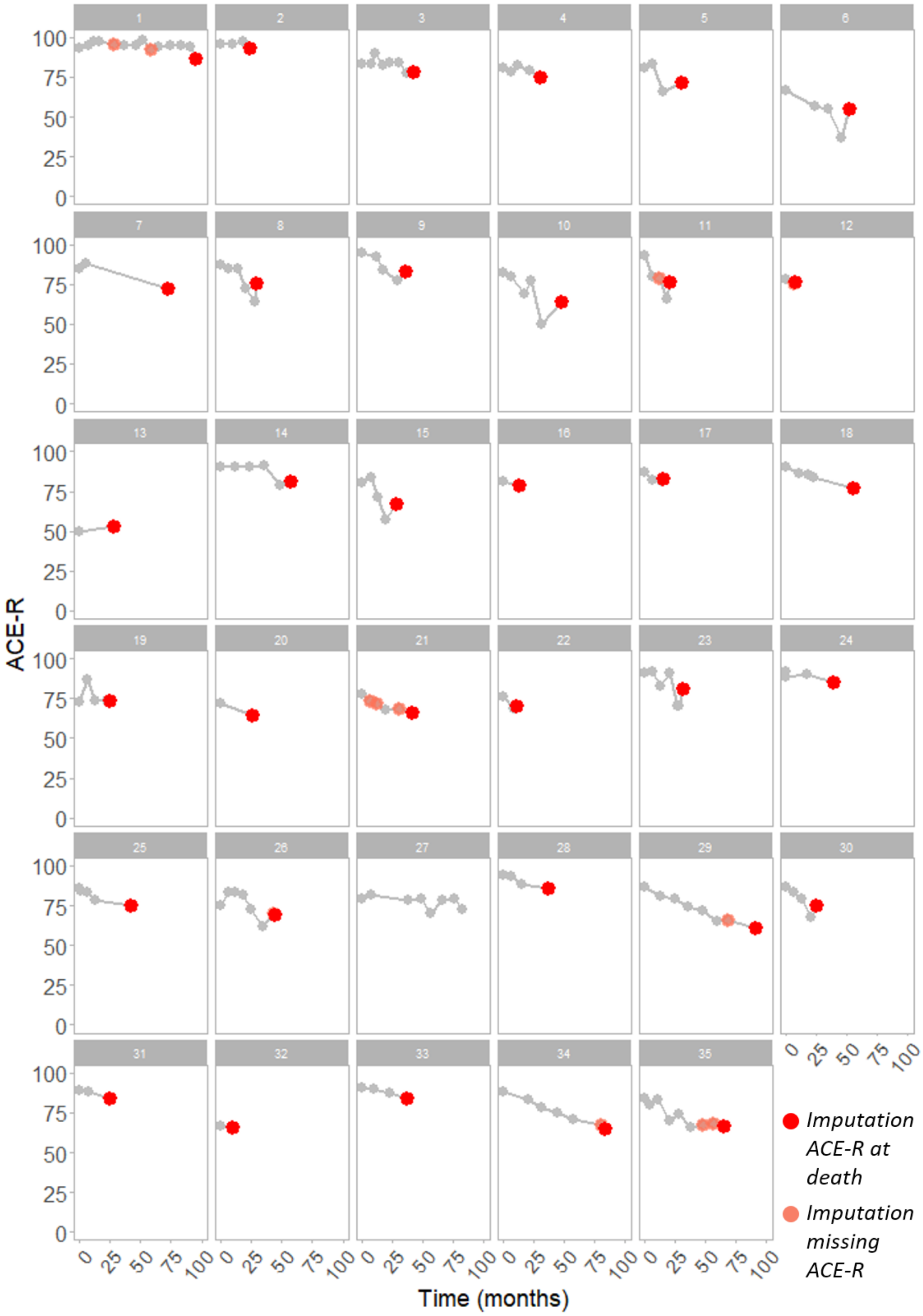

Supplementary fig. 4 *Longitudinal and imputed ACE-R for individual PSP patients. PSPRS and ACE-R accessed at visits to the clinic (grey points) were used to impute ACE-R values at death (red points) and at visits where only PSPRS were measured (pink points) for each of the 35 PSP patients. The x-axis show time from the first visit (0) to death in months. The order of patients (1-35) is commensurate with that of Figure 2. ACE-R – revised Addenbrooke's Cognitive Examination revised, PSPRS – Progressive Supranuclear Palsy Rating Scale*
