# Supplementary material for: Validation of the new pathology staging system for progressive supranuclear palsy
Source: Acta Neuropathol. 2021 Mar 28;141(5):787–9. doi: 10.1007/s00401-021-02298-z (PMC8043892; doi:10.1007/s00401-021-02298-z)
Supplement: Supplementary file 3 — Supplementary file3 (PDF 109 KB) [file 401_2021_2298_MOESM3_ESM.pdf]

Supplementary Methods

Title

Validation of the new pathology staging system for progressive supranuclear palsy

Authors

Mayen Briggs[1], Kieren SJ Allinson[1], Maura Malpetti[2,3], Maria Grazia Spillantini[2], James Benedict Rowe[2,3,4], Sanne Simone Kaalund[2,3]

[1] Cambridge University Hospitals NHS Foundation Trust and the Cambridge Brain Bank, CB2 2QQ

[2] Department of Clinical Neurosciences, University of Cambridge, Cambridge Biomedical Campus, Cambridge, CB2 0SZ

[3] Cambridge Centre for Parkinson-plus, University of Cambridge

[4] Medical Research Council Cognition and Brain Sciences Unit, University of Cambridge, UK, CB2 7EF

Corresponding author

Address: Department of Clinical Neurosciences, Clifford Allbutt Building, Hills Road, CB2 0AH, Cambridge, United Kingdom

Telephone: +44 1223762076

Email: ssk42@medschl.cam.ac.uk

Supplementary Methods

Case cohort

We include brain tissue donated to the Cambridge Brain Bank by 35 people with PSP who had participated in clinical studies at the Cambridge University Centre for Parkinson plus. Tissue was obtained under the ethically approved protocol for "Neurodegeneration Research in Dementia" (REC 16/WA/0240). Cases were selected based on a pathological diagnosis of PSP following the National Institute of Neurological Disorders and Stroke (NINDS) criteria, and availability of ante-mortem data including PSP rating scale [2] and the revised Addenbrooke's Cognitive Examination (ACE-R)[6]. The number of clinical review visits per patient where PSPRS and/or ACE-R were recorded ranged from 1 to 13, with median of 4.

The clinical diagnoses included probable PSP-RS (n=25), probable PSP-frontal (n=1), possible PSP-corticobasal syndrome (n=3), possible PSP-progressive gait freezing (n=1), suggestive of PSP-corticobasal syndrome (n=3), suggestive of PSP-speech/language disorder (n=1) and suggestive of PSP-Parkinsonism (n=1). For patients dying before the publication of the revised criteria for PSP [3] the new criteria were applied to clinical data at the last clinic visit (details in Gazzina et al. 2019 [1]) (Supplementary table 1, online resource).

Immunohistochemistry and pathological evaluation

In all cases neuropathological evaluation and staging was performed on the left hemisphere.

Following the proposed guidelines for PSP pathology staging [4] paraffin-embedded tissue blocks including the globus pallidus (GP), subthalamic nucleus (STN), striatum (STR), middle frontal gyrus (Fr), dentate nucleus and cerebellar white matter (DE/CB), and occipital cortex (Oc) were immunohistochemically stained for hyperphosphorylated tau, AT8 (AT8, MN1020, Thermo Scientific, USA). Cytopathologies were scored for the six regions on a 4 point scale from none (-) to severe (+++); *none* (-) was defined as no or single cell pathology for every 20th field of view under a 40X objective. Specifically, we evaluated neuronal and oligodendroglial cytopathology in the GP and DE/CB, neuronal cytopathology in the STN, and astrocytic cytopathology in STR, Fr and Oc. If neurofibrillary tangles were observed in the STR in addition to astrocytic tau, a rating of moderate or severe was given. Neuronal cytopathology included neurofibrillary tangles, diffuse cytoplasmic inclusions and tau positive threads. Oligodendrocytic cytopathology included coiled bodies, while astrocytic cytopathology included tufted astrocytes. Supplementary fig. 1 (online resource) provides a visual guide for the scoring system for each of the six regions.

Tissue blocks for pathological diagnosis were sampled according to NINDS standard guidance for neurodegenerative diseases from brainstem, subcortical and cortical areas and were evaluated for the initial pathological diagnosis of PSP and possible concomitant pathologies of amyloid beta (Clone 6F/3D, M0872, Dako, Denmark), alpha-synuclein (SA3400, Enzo life sciences, USA) and TDP-43 (TIP-PTD-P02, Cosmo Bio Co LTD, Japan), and vascular pathology.

Statistical analysis

Statistical differences in age at death and symptom duration were analysed by Kruskal Wallis test. Disease duration was calculated as time interval between symptom onset and death. Main effects of tau pathology scores on *ante mortem* clinical severity, as measured by the PSPRS and ACE-R scores at the last individual clinical visit, were analysed using one-way weighted means ANOVA. The reciprocal of time between testing and death was used as the weight, giving more weight to those data points with the shortest interval from test date to date of death. Then, we also imputed the PSPRS and ACE-R scores at death using multivariate imputation by chain equations as described in Malpetti et al. [5] (Supplementary fig. 3 and 4, online resource). In brief, we used the individual longitudinal data for PSPRS and ACE-R scores, and months from baseline to each follow-up visit to estimate missing scores for disease severity (PSPRS) and cognition (ACE-R), including scores at death. Patient reference numbers were included in the imputation to account for individual differences. Four patients had no PSPRS assessment, and PSPRS scores was imputed from the group mean adjusted for ACE-R. The imputed values at death where used in a one-way ANOVA to compare PSPRS and ACE-R between pathology stages. Main effects were considered significant at $p < 0.05$, we did not correct for multiple comparisons. All statistical analysis and graphical plots were carried out in RStudio (v. 4.0.2).

References

1. Gazzina S, Respondek G, Compta Y, Allinson KS, Spillantini MG, Molina-Porcel L, Guasp-Verdaguer M, Moftakhar S, Reich SG, Hall D, Litvan I, Hoeglinger G, Rowe JB (2019) Neuropathological validation of the MDS-PSP criteria with PSP and other frontotemporal lobar degeneration. bioRxiv 520510. doi: 10.1101/520510

2. Golbe LI, Ohman-Strickland PA (2007) A clinical rating scale for progressive supranuclear palsy. Brain 130:1552–1565. doi: 10.1093/brain/awm032

3. Höglinger GU, Respondek G, Stamelou M, Kurz C, Josephs KA, Lang AE, Mollenhauer B, Müller U, Nilsson C, Whitwell JL, Arzberger T, Englund E, Gelpi E, Giese A, Irwin DJ, Meissner WG, Pantelyat A, Rajput A, van Swieten JC, Troakes C, Antonini A, Bhatia KP, Bordelon Y, Compta Y, Corvol J-C, Colosimo C, Dickson DW, Dodel R, Ferguson L, Grossman M, Kassubek J, Krismer F, Levin J, Lorenzl S, Morris HR, Nestor P, Oertel WH, Poewe W, Rabinovici G, Rowe JB, Schellenberg GD, Seppi K, van Eimeren T, Wenning GK, Boxer AL, Golbe LI, Litvan I, Movement Disorder Society-endorsed PSP Study Group (2017) Clinical diagnosis of progressive supranuclear palsy: The movement disorder society criteria. Mov Disord 32:853–864. doi: 10.1002/mds.26987

4. Kovacs GG, Lukic MJ, Irwin DJ, Arzberger T, Respondek G, Lee EB, Coughlin D, Giese A, Grossman M, Kurz C, McMillan CT, Gelpi E, Compta Y, van Swieten JC, Laat LD, Troakes C, Al-Sarraj S, Robinson JL, Roeber S, Xie SX, Lee VMY, Trojanowski JQ, Höglinger GU (2020) Distribution patterns of tau pathology in progressive supranuclear palsy. Acta Neuropathol 140:99–119. doi: 10.1007/s00401-020-02158-2

5. Malpetti M, Passamonti L, Rittman T, Jones PS, Vázquez Rodríguez P, Bevan-Jones WR, Hong YT, Fryer TD, Aigbirhio FI, O’Brien JT, Rowe JB (2020) Neuroinflammation and Tau Colocalize in vivo in Progressive Supranuclear Palsy. Ann Neurol 88:1194–1204. doi: 10.1002/ana.25911

6. Mioshi E, Dawson K, Mitchell J, Arnold R, Hodges JR (2006) The Addenbrooke’s Cognitive Examination revised (ACE-R): A brief cognitive test battery for dementia screening. Int J Geriatr Psychiatry 21:1078–1085. doi: 10.1002/gps.1610
